# Advance care planning in primary care for patients with gastrointestinal cancer: feasibility randomised trial

**DOI:** 10.3399/BJGP.2021.0700

**Published:** 2022-06-28

**Authors:** Anne Canny, Bruce Mason, Jacqueline Stephen, Samantha Hopkins, Lucy Wall, Alan Christie, Richard JE Skipworth, Joanna Bowden, Louise Graham, Marilyn Kendall, Christopher J Weir, Kirsty Boyd

**Affiliations:** Primary Palliative Care Research Group, Usher Institute, University of Edinburgh, Edinburgh.; Primary Palliative Care Research Group, Usher Institute, University of Edinburgh, Edinburgh.; Edinburgh Clinical Trials Unit, Usher Institute, University of Edinburgh, Edinburgh.; Edinburgh Cancer Centre, Western General Hospital, Edinburgh.; Edinburgh Cancer Centre, Western General Hospital, Edinburgh.; Edinburgh Cancer Centre, Western General Hospital, Edinburgh.; Royal Infirmary of Edinburgh; honorary reader, Clinical Surgery, University of Edinburgh, Edinburgh.; NHS Fife, Fife; clinical research fellow, University of St Andrews, St Andrews.; Edinburgh Cancer Centre, Western General Hospital, Edinburgh.; Primary Palliative Care Research Group, Usher Institute, University of Edinburgh, Edinburgh.; Edinburgh Clinical Trials Unit, Usher Institute, University of Edinburgh, Edinburgh.; Primary Palliative Care Research Group, Usher Institute, University of Edinburgh, Edinburgh.

**Keywords:** advance care planning, anticipatory care planning, cancer, general practice, mixed methods, primary health care

## Abstract

**Background:**

Advance (anticipatory) care planning (ACP) requires discussions between patients and healthcare professionals about planning for future deterioration in health. ACP improves care coordination but uptake is limited and often deferred.

**Aim:**

To assess the feasibility and acceptability to patients, carers, and GPs of a primary care ACP intervention for people with incurable oesophageal, gastric, or pancreatic cancer.

**Design and setting:**

A 12-month feasibility randomised controlled trial (RCT) in a Scottish Cancer Network.

**Method:**

Patients aged ≥18 years starting palliative oncology treatment were randomised 1:1 to an ACP intervention or standard care. Patients in the intervention group received an oncologist letter supporting them to request a GP review along with a patient information leaflet about ACP. Pre-specified analyses with masking included trial recruitment and retention, ACP completion, and quality-of-life questionnaires (EuroQol EQ-5D-5L and ICECAP Supportive Care Measure) at baseline, 6, 12, 24, and 48 weeks. Qualitative interviews with purposive sampling explored patient, carer, and GP experiences.

**Results:**

Of 99 eligible participants (269 screened), 46% were recruited (*n* = 46) and randomised; 25 to intervention and 21 to control. By 12 weeks, 45% (*n* = 9/20) of the individuals in the intervention and 59% (*n* = 10/17) in the control group had a documented ACP plan. By 24 weeks, 30% (*n* = 14) had died; in the remaining participants quality of life was maintained at 24 weeks except for physical symptoms. Social norms associating ACP with dying were prevalent among 23 participants interviewed. No psychological or clinical harms were identified.

**Conclusion:**

An RCT of ACP for people with incurable cancer in primary care is feasible. Patient, carer, and GP attitudes and behaviours determined acceptability and timing of care planning.

## INTRODUCTION

Advance (anticipatory) care planning (ACP) is an internationally recommended approach to care of patients with deteriorating health.^1^ ACP involves hospital clinicians, GPs, and primary care teams talking with patients and families about the future, and making plans for when their health changes.^2^^–^^4^ Uncertainties are acknowledged and addressed, and proactive care plans agreed for potential health and care crises, and to prevent avoidable acute hospital admissions.^5^ ACP is particularly relevant for people with life-limiting illnesses such as incurable cancer, as well as those with progressive long-term conditions.

In the UK, patients with cancer have an average of five emergency admissions to hospital in the last year of life and the highest per person use of unscheduled care.^6^^,^^7^ Unplanned admissions disrupt continuity of care in the community and may lead to inappropriate investigations and interventions.^8^ People with incurable cancers may need such admissions because of illness severity, complexity, and complications.^9^ Growing evidence shows this group of patients with cancer value and benefit from early support in primary care as well as from oncology soon after diagnosis to improve quality of life, symptom management, and care coordination.^10^^–^^13^ Integration of oncology, specialist palliative care, and primary care has been recommended.^14^

This study aimed to evaluate the feasibility and acceptability of an early ACP intervention in primary care designed to trigger proactive discussions between patients and their GP leading to a documented advance care plan in the Scottish primary care electronic record (key information summary — advance [anticipatory] care plan [KIS-ACP]). A secondary objective was to empower and encourage patients and their family/carers to engage in an ACP process soon after starting palliative oncology treatment.

## METHOD

### Design

This was a 12-month feasibility randomised controlled trial (RCT) with integration of quantitative and qualitative data collected and analysed in parallel (ClinicalTrials. gov identifier: NCT03719716). Reporting is in line with the CONSORT extension for randomised pilot and feasibility trials.^15^^,^^16^

**Table:** How this fits in

Advance (anticipatory) care planning (ACP) has established benefits for patients with cancer and other serious illnesses but uptake remains low in many countries including the UK. Offering opportunities for people to discuss their priorities and preferences, and have these recorded, informs future care and reduces inappropriate interventions. Patients and families prefer ACP conversations with known community professionals and when not acutely ill. Identifying and responding to people’s preferred care planning style can facilitate early support and meaningful conversations about ACP in primary care.

Primary feasibility outcomes were the proportion of eligible patients recruited and randomised, and the number of patients in the intervention group who had an ACP review by their GP documented in a new or updated electronic record within 12 weeks. Health-related quality of life was the main patient-reported outcome measured at baseline, 6, 12, 24, and 48 weeks. Mapping patient journeys from diagnosis to 12-month survival or death and service use were secondary clinical outcomes. Qualitative interviews with purposive sampling explored patient, carer, and GP experiences (Supplementary Appendices S1 and S2).

### Sample size

The study planned to include 50 participants (25 per group) to provide acceptable precision in estimating feasibility outcomes. If the true conversion rate from screening to consent were 50%, a 95% confidence interval (CI) for the rate estimate would have width +/–9.8%. The CI width for the proportion of participants randomised to the intervention who made a GP appointment would lie between +/–11.8% and +/–19.6% across a range of true proportions (10% to 90%).

Qualitative research practice relies on the core principle of data saturation rather than the number of participants recruited.^17^ Purposive sampling was utilised to give sufficient range and depth of patient, carer, and GP perspectives until no new codes or themes emerged.^18^

### Setting and patients

Patients aged ≥18 years starting palliative oncology treatment for newly diagnosed incurable pancreatic or upper gastrointestinal (oesophageal or gastric) cancer at a Scottish regional cancer centre were eligible to participate. Exclusion criteria included people who were unfit for oncology treatment, chose supportive care, had another life-limiting condition, or with cognitive impairment that precluded informed consent, communication by telephone, questionnaire completion, or interview.

Initial eligibility screening happened at weekly multidisciplinary cancer team meetings (MDT). Oncology clinicians offered study information to eligible patients at their clinics. Interested patients consented to transfer their contact details to the research team. Participants gave informed consent before being recruited and randomised to either early support (intervention) or standard care (control) in a 1:1 ratio. Randomisation was stratified by diagnostic group (pancreatic or gastrointestinal cancer) and health board. The allocation sequence was created by a database programmer at Edinburgh Clinical Trials Unit using computer-generated pseudo-random numbers with random permuted blocks and was concealed by use of a centralised web-based randomisation system (REDCap).

### Intervention

Participants in the intervention arm received a personal letter developed with a patient–public involvement (PPI) group and signed by their oncologist to help them make an appointment to discuss ACP with their GP. The Healthcare Improvement Scotland patient information leaflet on ACP (known as ‘anticipatory care planning’ in Scotland) was enclosed with this letter and the study information booklet.^19^ General practices received the same documents plus professional information about ACP,^20^ a copy of the NHS Scotland ‘RED-MAP’ communication framework (2019),^21^ and a request to consider starting ACP and complete an electronic ACP plan. Control group participants received standard care.

### Quantitative instruments and data analysis

Research team clinicians screened electronic health records for all patients reviewed at the cancer meetings and records of patients subsequently assessed in oncology clinics to document exclusion reasons. All patients agreeing to speak to a researcher were consented and randomised.

Study clinicians recorded key events including withdrawal because of poor health or death. GP practices provided service use data including dates of GP contacts. All participants completed validated quality-of-life measurements at baseline, 6, 12, 24, and 48 weeks: EuroQol EQ-5D-5L and ICECAP Supportive Care Measure (ICECAP-SCM).^22^^,^^23^ The ICECAP-SCM has seven wellbeing domains but the study’s PPI group recommended removal of the final question referring to preparation for death as potentially distressing for people starting cancer treatment. Scores for individual domains were reported as summative scores could not be calculated. The booklets also included the CollaboRATE questionnaire, which is a simple measure of shared decision making suited to primary care.^24^ Intervention group questionnaire booklets had two supplementary questions asking if the letter helped obtain a GP appointment within 12 weeks that led to discussion about ACP.

The researcher collated all data for analysis into spreadsheets using unique participant identifiers. Two clinical researchers, who were masked to trial arm, extracted and graded (high, medium, or low) ACP content in the electronic urgent care records (KIS) for participants in one health board each. A similarly masked, academic clinician completed an independent review of all KIS records from both the health boards. A high-quality KIS had a timely record of key events, informative treatment, and care plans, and documented cardiopulmonary resuscitation status.

Quantitative data analysis followed a pre-specified analysis plan (Supplementary Appendix S2) by the trial statistician using SAS (version 9.4) with participants identified solely by study code number to maintain masking. Analysis consisted of descriptive statistics with baseline demographic and clinical data summarised overall and by treatment arm. Feasibility outcomes were calculated as a proportion with exact 95% CIs. Overall study retention rates and differences between intervention/control groups with 95% CIs were estimated. Baseline data and feasibility outcomes were analysed by stratification factor. Patient-reported outcomes (EuroQol EQ-5D-5L, ICECAP-SCM, and CollaboRATE), service use, and time-to-event outcomes were summarised by randomised group and overall at each time point. These data were stored in a secure university server.

### Qualitative data generation and analysis

A purposive subsample of participants and their carers/family members from both groups based on diagnosis, demography, health board, and treatment arm participated in semi-structured interviews at weeks 6–8 and 20–22. Participants chose a joint or individual interview in their own home or at their local cancer clinic. A pre-specified topic guide facilitated exploration of treatment and care experiences, views about the intervention and survey instruments, and perceptions about trial participation. With patient consent, their GP gave a 20–30 minute telephone interview with the outline sent in advance. Interviews covered professional perceptions of the intervention and ACP processes in primary care including possible improvements.

Interviews with patients and carers/family members were analysed as dyads and addition of a GP interview constituted a triad. Interviews were recorded with an encrypted digital recorder, transcribed, and anonymised with a unique code. Transcripts were uploaded to NVivo (version 12) then coded with a framework based on the interview topic guides. Data were analysed using a thematic approach, within and across dyads and triads, and longitudinally in line with established narrative analysis methods.^25^

### Data integration and interpretation

Quantitative and qualitative datasets were analysed separately then integrated to generate a more comprehensive understanding of ACP in primary care for people with incurable cancers.^26^ Expert steering group meetings and research team discussions considered and managed areas of conflict, agreement, or enrichment in these data. Study findings were informed by the theory of planned behaviour to help describe and interpret ACP processes from patient/carer and GP perspectives.^27^ The theory of planned behaviour model has been applied extensively in healthcare contexts including hospice care and cancer screening.^28^^,^^29^

## RESULTS

**Figure 1.**
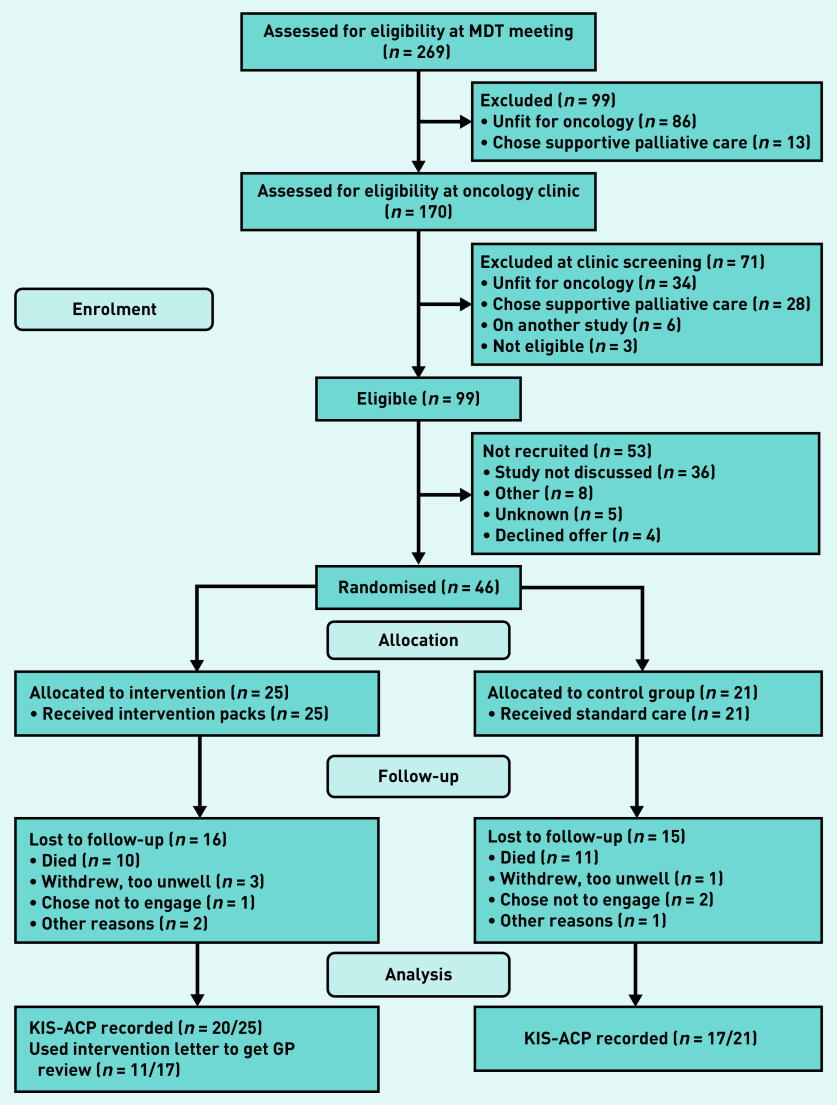
*CONSORT (2010) diagram of study participant flow, adapted for this study.^16^ KIS-ACP = key information summary — advance (anticipatory) care plan. MDT = multidisciplinary team.*

Participants were recruited from 1 January 2019 to 3 March 2020 and followed for up to 12 months until 31 August 2020. In March 2020, new recruitment stopped because of COVID-19 and some participants did not have enough time during the study to complete the final questionnaires. Of 269 screened patients, many (*n* = 170/269; 63%) had advanced disease and/or poor health at diagnosis so did not start oncology treatment or opted for palliative care. A further 53 eligible participants were not recruited mostly because the study was not offered to them by clinicians. This left 46 (46%) to be randomised, with 25 allocated to the intervention arm and 21 to the control arm (Figure 1 and Supplementary Appendix S3.1).

Relatively few eligible patients and recruitment delays meant 22 participants enrolled too late to reach the 48-week data point. Nine participants remained in the study and six completed the final questionnaires. The main feasibility outcome (a new or updated KIS-ACP) was measured at week 12 and assessed for all 46 participants.

Recruited patients were younger, having a median age of 65 years (range 59–71 years). Fewer recruited participants had pancreatic cancer (*n* = 12/46; 26%) because of its poorer prognosis and co-recruitment to another study. Table 1 shows the baseline characteristics of all screened patients (Supplementary Appendices S3.2–S3.4). Participants were keen to participate despite their diagnosis and debilitating symptoms or treatment side effects. Many wanted to share their experiences and improve care for future patients. Study retention was high in both groups with most withdrawals because of death or deteriorating health (Figure 1 and Supplementary Appendices S3.5 and S3.6). By 24 weeks, 30% (*n* = 14) of participants had died. At week 6, 64% (*n* = 28/44) returned their postal questionnaires. The researcher changed to collecting questionnaire responses by telephone in the final 8 months because of COVID-19 restrictions with a 66% (*n* = 21/32) overall completion rate at week 24.

**Table 1.** Baseline characteristics of all screened patients

**Characteristic**	**All (*n* = 269)**	**Not eligible (*n* = 170)**	**Eligible but not randomised (*n* = 53)**	**Randomised (*n* = 46)**
**Age, years**				
Mean (SD)	71 (11)	74 (11)	68 (10)	65 (9)
Median (Q1–Q3)	72 (64–79)	76 (68–83)	68 (62–74)	65 (59–71)
Minimum, maximum	42, 98	42, 98	45, 92	45, 80

**Sex,** ***n* (%)**				
Male	175 (65)	113 (66)	31 (58)	31 (67)
Female	94 (35)	57 (34)	22 (42)	15 (33)

**Cancer diagnosis,** ***n* (%)**				
Oesophagus/junctional	119 (44)	68 (40)	24 (45)	27 (59)
Stomach	44 (16)	32 (19)	5 (9)	7 (15)
Pancreas	106 (39)	70 (41)	24 (45)	12 (26)

**Main disease group,** ***n* (%)**				
Upper gastrointestinal	163 (61)	100 (59)	29 (55)	34 (74)
Pancreas	106 (39)	70 (41)	24 (45)	12 (26)

**First diagnosis,** ***n* (%)**				
Yes	261 (97)	166 (98)	50 (94)	45 (98)
No — relapsed disease	8 (3)	4 (2)	3 (6)	1 (2)

**Disease extent — metastatic,** ***n* (%)**				
Yes	169 (63)	100 (59)	35 (66)	34 (74)
No	100 (37)	70 (41)	18 (34)	12 (26)

**Initial oncology treatment plan made at MDT review,** ***n* (%)**				
Chemotherapy	156 (58)	68 (40)	44 (83)	44 (96)
Radiotherapy	12 (4)	2 (1)	8 (15)	2 (4)
Other	101 (38)	100 (59)	1 (2)	0 (0)

*MDT = multidisciplinary team. Q = quartile.*

A total of 23 patients participated in semi-structured interviews, often with a carer. Ten case-linked GPs were interviewed. Table 2 provides details of the individual, dyad, and triad interviews. While completing questionnaires by telephone, participants often talked about their experiences. With verbal consent, 21 of these storied accounts were recorded as field notes, providing further rich data for integration with formal interview data.

**Table 2.** Number of first, second, and dyad/triad interviews

**Category**	**Patient and carer**	**Patient alone**	**Carer alone**	**GP**	**Total**
**First interview**	16	7	1	10	34
Intervention	9	4	1	6	20
Control	7	3	—	4	14

**Second interview**	13	3	3	—	19
Intervention	8	2	2^a^	—	12
Control	5	1	1	—	7

**Total interviews**	29	10	4	10	53

a *Included one carer in a bereavement interview.*

### Feasibility outcomes

Feasibility outcomes from patient clinical data found a new or updated KIS for 83% (*n* = 20/24) in the intervention group and 85% (*n* = 17/20) in the control group by week 12. An ACP plan was included in the KIS summaries of 45% (*n* = 9/20) of the intervention and 59% (*n* = 10/17) of the control group (Table 3 and Supplementary Appendices S3.7 and S3.8). The overall KIS-ACP quality was similar between groups, but almost half of the ACP plans were rated as poor by experienced clinicians. Medium-to high-quality ACP plans were mostly completed in the last 2–3 months of life. The letter helped 65% (*n* = 11/17) of participants answering the question about obtaining a GP appointment to get one. All 13 patients in the intervention group interviewed took the letter to their GP practice.

**Table 3.** Outcomes analysis of GP urgent care records (KIS), ACP plan content, and quality

**Intervention feasibility outcomes**	**All (*n*= 46)**			**Intervention (*n*= 25)**			**Control (*n* = 21)**		
		
	***N***	***n* (%)**	**95% CI**	***N***	***n* (%)**	**95% CI**	***N***	***n* (%)**	**95% CI**
**Eligible patients who were randomised**	99	46 (46)	36.4 to 56.8	N/A	N/A	N/A	N/A	N/A	N/A

**New or updated KIS in patient clinical record**	44	37 (84)	69.9 to 93.4	24	20 (83)	62.6 to 95.3	20	17 (85)	62.1 to 96.8

**Clear ACP plan included in patient KIS**	37	19 (51)	34.4 to 68.1	20	9 (45)	23.1 to 68.5	17	10 (59)	32.9 to 81.6

**Letter helped me get a GP appointment^a^**	N/A	N/A	N/A	17	11 (65)	38.3 to 85.8	N/A	N/A	N/A

**Clinician assessment of KIS-ACP quality^b^**	37			20			17		
High		7 (19)	N/A		2 (10)	N/A		5 (29)	N/A
Medium		12 (32)	N/A		7 (35)	N/A		5 (29)	N/A
Low		18 (49)	N/A		11 (55)	N/A		7 (41)	N/A

a *Included in denominator if participants had provided any response (yes/no) to the question.*

b *For KIS-ACP quality, post hoc clinical review changed the grading of KIS-ACP assessments (n = 4) after the quantitative analysis report had been issued. ACP = advance (anticipatory) care planning. KIS = key information summary. KIS-ACP = key information summary — advance (anticipatory) care plan. N/A = not applicable.*

Relationships between GPs and patients, perceived busyness of the practice, and appointment systems had an impact on both groups. GPs thought the intervention letter a useful prompt to consider earlier engagement with patients. Documented future care plans were valuable but required a difficult, delicate balance between encouraging patients to engage in challenging conversations and respecting individual wishes.

Attitudes and perceptions of individual patients, carers, and GPs determined subsequent ACP outcomes and the interviews revealed four styles of care planning behaviour:

- early patient/carer planners started making future care planning decisions around the time of diagnosis, and GPs were proactive in initiating ACP conversations;
- evolving patient/carer planners began planning ahead as the disease progressed, and GPs initiated ACP when prompted by patients/carers, oncology clinicians, or district nurses;
- late patient/carer planners only made plans towards the terminal phase of illness, and GPs initiated ACP late in the disease trajectory; and
- non-planners did not make care plans.

Early patient planners were uncommon and most had worked in health care. Many participants had not engaged in ACP by second interview. From 10 GPs interviewed, three intervention and one control group GP were early planners. Interactions between people with different planning styles emerged as more important than the intervention in determining outcomes; shown here in the Results and in Supplementary Appendix S4.

#### Interview triad — early planner patient, carer, and GP

*‘Our GP took the bull by the horns right from the start. She asked me about end of life and if I wanted resuscitated as soon as I was diagnosed, and I said no.’*

(Control group patient [23P], second interview, 6 February 2020)

*‘Some patients find it very difficult to engage with us as GPs or our primary health care team if they are very much fixated on what can oncology offer. Sometimes, we as GPs feel that patients actually haven’t got to that stage. There is a small group who cling on for that last bit of hope. So, that is led by the patient, unfortunately.’*

(GP interview for control patient 23P, 6 February 2020)

#### Interview triad — non-planner patient, carer, and early planner GP

*‘It’s hard to say what he said. Something about this is about what you want towards the end of your life. But we’re just no feeling at that stage yet. I’ll sort of cross that bridge when I come to it.’*

(Intervention group patient [43P], first interview, 10 April 2020)

*‘He has a KIS, yes. We discussed ACP and he was meant to get back to me about it, but he hasn’t yet. And that was when all the COVID stuff came up, so that’s been disrupted.’*

(GP interview for intervention patient 43P, 26 June 2020)

#### Interview triad — non-planner patient, carer, and late planner GP

*‘I handed it into the receptionist. And I’ll be honest with you, I did’nae* [didn’t] *really need the doctor, so I’ve never really bothered him. He’s* [GP] *never been in touch about the cancer. Looking ahead with the cancer, I’m nae* [not] *worried. Mine’s no* [not] *malignant, and it’s no* [not] *spread. If I get another 10 years, I’ll be quite happy.’*

(Intervention group patient [26P], first interview, 6 December 2019)

*‘I’ve never given that a thought. Well, I’m telling a lie there; I have thought about it and basically, I wouldn’t want resuscitated. I wouldn’t mind being asked that question, but I’ve never been asked. I feel that if my quality of life was over, I’d rather just be.’*

(26P, second interview, 3 March 2020)

*‘The KIS has been activated but it simply says a new diagnosis of atrial fibrillation. We don’t have a standard policy on palliative care. We are expected to make all of them a KIS with a palliative care summary. In reality, we have a lot of patients who you could call palliative, but who are very well and have a full, active, unrestricted life.’*

(GP interview for intervention patient 26P, 3 March 2020)

**Figure 2.**
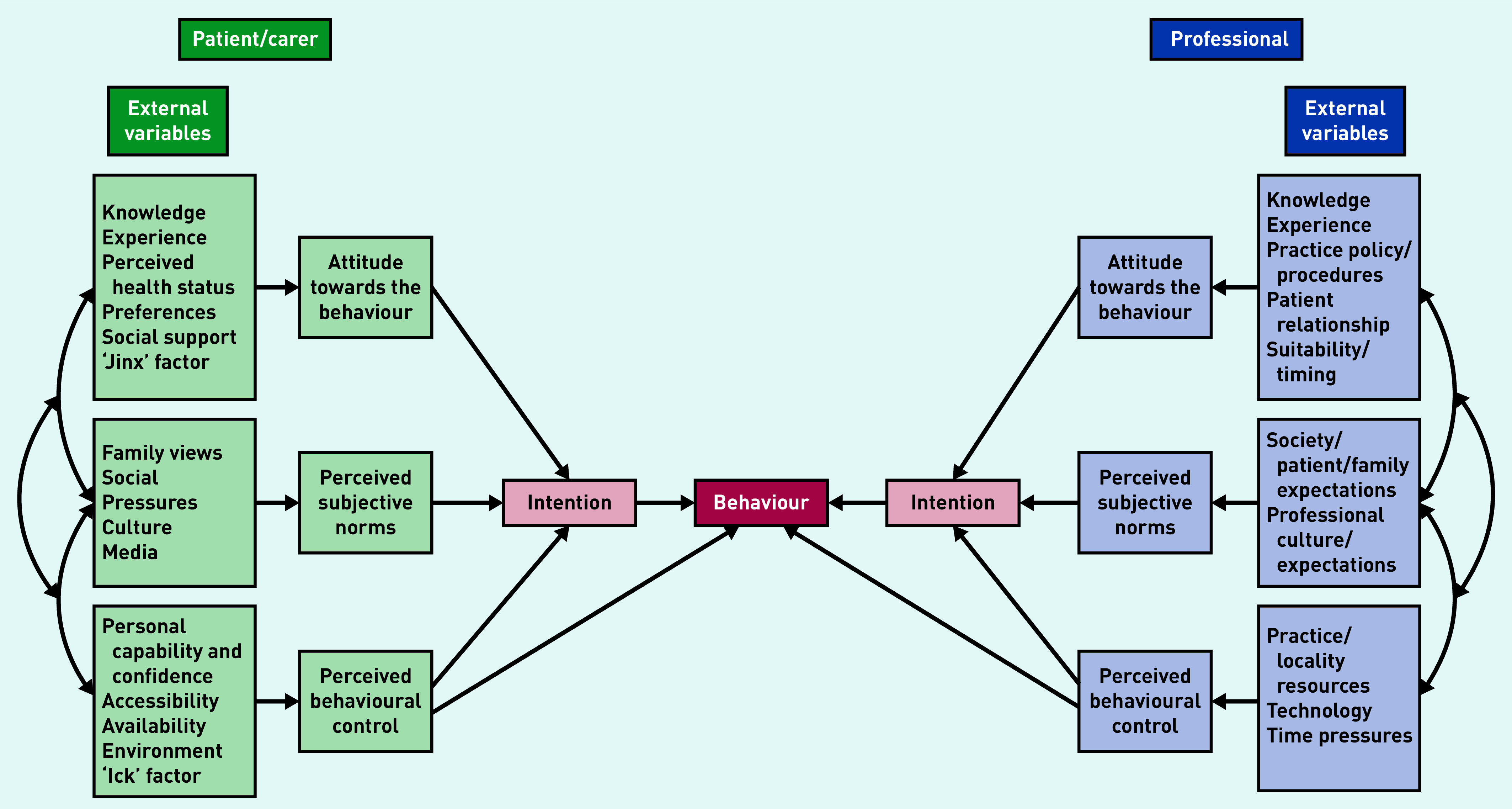
*Theory of planned behaviour applied to advance (anticipatory) care planning.*

Attitudes towards ACP and strongly held beliefs about its association with cardiopulmonary resuscitation and planning for the end of life conflicted with living well in the present, maintaining normality, and perceived harms of planning ahead too soon. Public and professional information about ACP being an ongoing process of preparing for the future well before the last phase of life had little impact on prevailing social norms about fighting cancer and maintaining hope. With few exceptions, ACP was reactive and triggered by changes in the patient’s health or cancer treatment plan. Pressure of time and the drive to complete resuscitation forms (DNACPR, do not attempt cardiopulmonary resuscitation) affected perceived behavioural control among GPs. Figure 2 illustrates factors underlying these ACP behaviours.

### Patient-reported outcomes — quality of life

Patients in both groups maintained a good quality of life despite their poor prognosis and the impact of treatment side effects. Baseline EuroQol EQ-5D-5L had a median Health Index score of 0.72 (Q1–Q3, 0.66–0.81) and visual analogue scale (VAS, 0–100) median score of 75 (Q1–Q3, 65.0–85.0). Scores remained stable to week 24 where the median Health Index was 0.71 (Q1–Q3, 0.55–0.77) and VAS score 70 (Q1–Q3, 60.0–85.0) (Supplementary Appendix S5.1).

Patients recorded high scores on five ICECAP-SCM wellbeing measures up to 24 weeks, shown as a range of scores: ‘Having a say most of the time’ (88%–100%); ‘Being with people who care about you most of the time’ (80%–97%); ‘Emotional suffering sometimes or rarely’ (85%–100%); ‘Able to maintain dignity most of the time’ (92%–100%); and ‘Being supported most of the time’ (93%–100%).

Scores for the ICECAP-SCM domain ‘Experiencing physical discomfort often or always’ (28%–50%) suggested most patients suffered increasing physical symptoms over time (Supplementary Appendix S5.2).

Interviewed patients shared experiences of living with cancer, and felt well supported with cancer treatment and involved in all aspects of decision making reflected in CollaboRATE scores from both groups. Patients recorded a top score for ‘Every effort was made to involve me’: baseline (65%), week 6 (59%), week 12 (48%), and week 24 (59%) (Supplementary Appendix S5.3). Trying to maintain normality and balance being pain free while minimising analgesic use and side effects were important considerations:

*‘It’s amazing because these are all pretty serious things and yet I’ve felt good and I’ve felt healthy enough and I’m eating well. I feel quite positive although there are the odd days where I feel a bit anxious and things.’*

(Control group patient [42P], fieldnotes, 31 July 2020)

*‘Well, if I could just get to the bottom of this back pain that makes me feel sick, I’d be absolutely fine. I can take the immediate release* [opioid] *any time I feel it coming on. It’s trial and error.’*

(Intervention group patient [38P], second interview, 23 July 2020)

### Clinical and service use outcomes

Service use by both groups appeared broadly similar. Patients in the intervention group spent more days in hospital (median 14.0 days [Q1–Q3, 5.0–24.0] versus 6.0 days [Q1–Q3, 3.0–11.0]). Home deaths appeared higher in the intervention group (53% versus 29%) although numbers were small. Median time from multidisciplinary cancer meeting to first GP contact was shorter in the intervention group but there was substantial overlap between groups indicating diversity of experiences among participants (7.3 weeks [Q1–Q3, 3.6–8.9] versus 13.1 weeks [Q1–Q3, 2.6–19.4]). GP visits or contacts were similar; median 8.0 (Q1–Q3, 3.0–11.0) through to the last week of life (Supplementary Appendices S5.4 and S5.5).

Cancer treatment stopped around 3 months before death in both groups. Of 46 participants, 37% (*n* = 17) received specialist palliative care, with later referral in the intervention group (26.4 weeks [Q1–Q3, 9.1–48.4] versus 11.6 weeks [Q1–Q3, 3.7–22.4]). Other time-to-event data were similar for all participants. The median survival from cancer review meeting was 30.6 weeks (Q1–Q3, 17.0–42.9), and a KIS was started or updated after a median of 18.6 weeks (Q1–Q3, 7.6–42.9) (Supplementary Appendices S5.4 and S5.5). A total of 29 (63%) patients died during the study, all from cancer or its complications (Supplementary Appendix S5.4). No psychological or clinical harms were identified.

## DISCUSSION

### Summary

This study found patients with incurable oesophageal, gastric, or pancreatic cancers starting palliative oncology treatment can be recruited to an ACP trial in primary care with good retention rates even though one-third died within 6 months. An intervention letter from oncology was acceptable to patients but did not trigger more ACP discussions leading to documentation of a clinically relevant ACP. The findings of this study offer insights into how people navigate incurable cancer and cancer treatment by maintaining normality; reflected in high quality-of-life scores and personal accounts. Interview dyads/triads illuminated an evolving dialogue between patients, family carers, and GPs about care planning as the patient’s health deteriorated. Where care planning styles aligned or GPs could broach thinking about the future linked to clinical events, meaningful conversations often followed.

### Strengths and limitations

This study has several strengths. This feasibility trial successfully evaluated an ACP intervention designed with local cancer clinicians, GPs, and public–patient representatives, and its implementation in routine clinical practice.^30^ Retention was good, with death or deterioration the main reasons for withdrawal. Validated questionnaires with good response rates measured quality of life and decision making about palliative cancer treatments. Health service use and time-to-event data mapped care pathways for a rapidly deteriorating patient group. Qualitative interview data generated in parallel were integrated with quantitative data for reporting.^31^ Longitudinal, multi-perspective interviewing was used to generate rich data about underlying attitudes, beliefs, social norms, and external factors making earlier ACP discussions in primary care possible but complex and individual.^32^

There were also study limitations. Many screened patients were not fit for cancer treatment or opted for palliative care. This slowed recruitment and meant follow-up to week 48 was not possible for participants joining the study later on. COVID-19 exacerbated difficulties recruiting GPs and obtaining primary care service use data. The pandemic may have had an impact on KIS completion and ACP quality through pressure to complete more plans that directed care in the event of COVID-19 infection. Excluding the final ICECAP-SCM wellbeing question on PPI advice prevented summative scoring across all domains. This study took place in a single cancer centre serving two Scottish health boards and participants did not represent ethnically diverse backgrounds, limiting generalisability.

### Comparison with existing literature

GPs have an important, valued role in providing care coordination for people with incurable cancers, along with general palliative care support, as a patient deteriorates.^13^^,^^33^ In this study, such care was evident particularly in the final phase of illness. Patients appreciated their cancer care and felt involved in treatment decision making but collaborative approaches to ACP between oncology services and primary care were lacking. Systematic reviews of ACP implementation studies have found that effective ACP programmes focus on individualised discussions over time and target multiple stakeholders to deliver integrated ACP.^34^ ACP intervention studies in primary care are underdeveloped compared with other settings and lack cohesive structures.^35^

Social norms associating ACP with death and dying, and fears about consequences of discussing them, were almost universal among participants. Research exploring end-of-life communication within families has shown how such normative values create multiple barriers to open dialogue including belief in positive thinking and protective buffering.^36^ Many people in the current study exhibited fluid and oscillating ‘dual dialogue’ or double awareness; the capacity of individuals to live as normally as possible while also being aware of the inevitability of death.^37^^,^^38^ This concept underpins successful early palliative care interventions in oncology out-patient services that support patients and family carers to gradually engage and cope with both living life well and acknowledging dying.^12^^,^^37^

The COVID-19 pandemic highlighted harms associated with policy drives to record advance treatment plans, including cardiopulmonary resuscitation status, at the expense of meaningful conversations about what matters to people at risk of deteriorating health.^39^ Shifting professional and public understanding away from ‘planning for dying’ towards broader, values-based concepts of ACP in the UK and internationally is increasingly accepted as best practice.^1^^,^^40^^,^^41^ Patient–public information and education about ACP is becoming more widely available and aims to normalise ACP by empowering people to have these important conversations with their family, friends, and clinicians.^42^^–^^44^ Primary care teams can be supported better to embed ACP in routine care, and start earlier conversations about serious illness, coping with uncertainty, and preparing for future changes in a person’s health by using resources developed by primary care leads.^35^^,^^45^ Electronic care coordination systems for ACP in primary and secondary care are being developed to be accessible, readily updated by all key professionals, and clinically relevant.^46^

### Implications for research and practice

Progression to a full trial of early ACP in primary care with people with incurable cancers is feasible in terms of patient recruitment and retention, and this study suggested a 24-week intervention may be sufficient. Longer follow-up remains valuable for secondary outcomes such as quality of life and health service use. People living with the challenges of incurable cancer and palliative oncology treatments were keen to participate in research and improve care for others. Timing and acceptability of ACP depended on perceptions and attitudes of patients, carers, and GPs. Understanding these behaviours is already informing future ACP research and clinical practice through the 2021 national toolkit for ACP in Scotland and development of a new digital platform that addresses current limitations of the KIS system. Primary care has a central role in ACP but collaborative models with oncology merit investigation. Public engagement campaigns should continue to promote wider discussions about future care and highlight benefits of planning for changes in health to help patients and carers navigate health and care crises. Values-based health care prioritises talking about what matters to a person; reflected in recent professional education on meaningful conversations about ACP.

## Data

All datasets are archived at the University of Edinburgh and available from the authors on reasonable request. The full statistical analysis report and additional qualitative data are provided in the Supplementary Data.
